# Leflunomide-induced pulmonary arterial hypertension in a young man with nephrotic syndrome: A case report

**DOI:** 10.3389/fphar.2022.992457

**Published:** 2022-10-17

**Authors:** Xiaoqin Luo, Jiang Li, Sisi Chen, Jun Luo

**Affiliations:** Department of Cardiology, The Second Xiangya Hospital of Central South University, Changsha, Hunan, China

**Keywords:** pulmonary arterial hypertension, leflunomide, nephrotic syndrome, tadalafil, macitentan

## Abstract

Drug-induced pulmonary arterial hypertension (PAH) has been widely reported but PAH caused by leflunomide is very rare. Here we report the case of a young man with nephrotic syndrome treated with leflunomide for 5 years before being admitted to our hospital for dyspnea. After discontinuing leflunomide treatment for 4 months, both the dyspnea and pulmonary artery systolic pressure improved. Right heart catheterization showed in a significant decrease in pulmonary vascular resistance and pulmonary artery pressure 4 months later. Because persistent PAH can lead to right heart failure and even death, identifying and excluding the risk factors is critical; discontinuing leflunomide until a definite cause is identified is highly recommended.

## Introduction

In pulmonary arterial hypertension (PAH), pulmonary arterial pressure is elevated while pressure in the left atrial and pulmonary veins is normal. More precisely, PAH is defined as mean pulmonary arterial pressure (MPAP) ≥ 25 mmHg, pulmonary arterial wedge pressure (PAWP) ≤ 15 mmHg, and pulmonary vascular resistance (PVR) ≤ 3 woods. PAH can be idiopathic, heritable, or associated with different conditions such as connective tissue diseases (CTD), human immunodeficiency virus (HIV) infection, and drugs or toxins (Galiè et al., 2016). Right heart catheterization (RHC) is the gold standard for diagnosing PAH. Use of the immunosuppressant leflunomide was shown to be associated with PAH in various diseases (Martinez Taboada et al., 2004; Alvarez et al., 2012; Coirier et al., 2018; Palasset et al., 2021). The duration of leflunomide usage ranged from 7 months to 120 months, with dosage ranged from 10 mg to 20 mg. One article reported that a woman with rheumatoid arthritis developed pulmonary hypertension after taking leflunomide for 2 years, with a mean pulmonary arterial pressure of 63 mmHg (MPAP) and pulmonary vascular resistance (PVR) of 19.9 wood (Alvarez et al., 2012). However, there have been few reports of nephrotic syndrome (NS) associated with PAH (Du et al., 2004), especially NS treated by leflunomide. Here we report a case of severe PAH in a young male patient with NS who was treated with leflunomide for 5 years. This case suggests that long-term use of leflunomide can lead to PAH.

## Case report

A 22 -year-old man with a 14-year history of NS was admitted to our hospital for dyspnea after exertion or walking 100 m that had persisted for 3 months. He complained of panting after physical activity, which was accompanied by chest pain that could be relieved by resting for several minutes. In 2008, he was admitted to hospital because of general edema. Renal biopsy showed mesangial hyperplasia with focal segmental sclerosis, and the edema was resolved after the first admission with treatment of prednison. Later, recurrent proteinuria occurred and it cannot be controled by adjustment of prednison, therefore, leflunomide was added in 2017 to suppress immunity and had been taking it until now. His medication included prednison 15 mg once daily (QD), with a gradual reduction in dosage to 2. 5 mg QD; leflunomide 20 mg QD from 2017 to March 2021, then 10 mg QD for 1 year; and telmisartan 40 mg QD from 2017 to the present. Upon physical examination, oxygen saturation in the extremities was around 90% and pulmonary second heart sound was loud.

**FIGURE 1:**
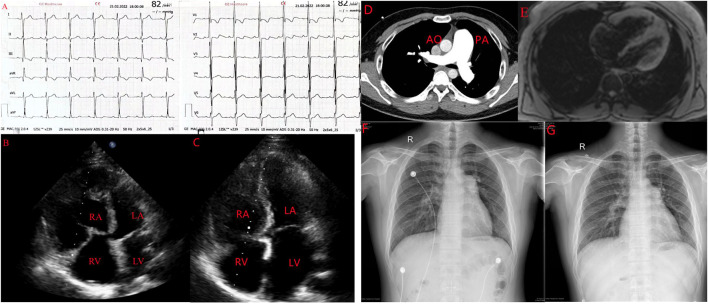
A case of leflunomide-induced PAH in a young male patient with nephrotic syndrome. **(A)**. Electrocardiograph showing signs of RV hypertrophy. **(B)** Echocardiogrph showing enlargement of the right side of the heart. **(C)**. Enlarged right side of the heart pressing on the LV to form a D sign. **(D)**. Computed tomography pulmonary angiography showing the ratio of the diameter of the dilated PA to the diameter of the aorta greater than **(E)**. Cardiac magnetic resonance showing dilation of the right side of the heart, consistent with pulmonary hypertension-induced cardiac changes. AO, aorta; LA, left atrium; LV, left ventricle; PA, pulmonary artery; RA, right atrium; RV, right ventricle. **(F,G)** Comparison of PA diameter before suspension of leflunomide and after suspension of leflunomide.

There was no jugular vein tension, no hepatic jugular reflux sign, and the apex beat was located 0.5 cm out side the fifth intercostal space of the left clavicle center. Blood gas analysis showed that arterial oxygen partial pressure was 64.60 mmHg (normal: 83.0–108.0 mmHg) and arterial oxygen saturation was 92. 8% (normal: 93–97%). N- terminal pro-brain natriuretic peptide (NT-proBNP) was 1406 0.0 pg/ml (normal: 0–450 pg/ml), which is outside the normal range. In terms of rheumatoid factors, the patient was positive for anti-histone antibodies and negative for rheumatoid factors, antinuclear antibodies, antineutrophil antibodies, antiphospholipid antibodies, vasculitis factors, and HIV. The maximum distance in the 6-min walk test was 409 m. An electrocardiogram revealed sinus rhythm with signs of right ventricular hypertrophy such as right axis deviation (>90°), R/S in V1 > 1, and R in V1+S in V5 > 1.2 mv (Figure 1A). An echocardiogram showed a right ventricle (RV) diameter of 52 mm, right atrium (RA) diameter of 38 mm, pulmonary artery (PA) diameter of 28 mm, inferior vena cava diameter of 14 mm, respiratory collapse rate <50%, and tricuspid regurgitation velocity of 5. 8 m/s. The estimated pulmonary systolic artery pressure (PASP) was 148 mmHg. A preliminary diagnosis of pulmonary hypertension (PH) was made and PH of the left heart was excluded (Figure 1B). Chest X-ray and computed tomography pulmonary angiography suggested PA distension and dilatation of the right heart; there was no evidence of thromboembolic and restrictive pulmonary diseases (Figure 1D) and no signs of portopulmonary hypertension (PoPH) in the abdominal ultrasound. Cardiac magnetic resonance findings were consistent with PH-related cardiac changes (Figure 1E). Based on these results and the patient’s medical history, we excluded 2/3/4 PH and CTD-, congenital heart disease-, and HIV-related PAH and PoPH. We performed RHC and the results showed that right atrial pressure was 11 mmHg, PASP was 153 mmHg, PADP was 53 mmHg, MPAP was 84 mmHg, PAWP was 10 mmHg, PVR was 24.7 woods, and the adenosine vasodilator test was negative.

After suspending leflunomide and treating the patient with macitentan and tadalafil for 4 months, we reviewed the patient‘s exercise tolerance and hemodynamic indices. The 6–min walking distance was improved to 480 m. NT-ProBNP was decreased to 110 pg/ml. The echocardiogram showed an RV diameter of 43 mm, RA diameter of 38 mm, PA diameter of 27 mm, tricuspid regurgitation velocity of 4 0.7 m/s, and estimated PASP of 97 mmHg (Figure 1C). Compared with the first X-ray, the PA diameter was obviously reduced (Figures 1F,G). Additionally, RHC showed a MPAP of 70 mmHg and PVR of 9.5 woods, which were both lower than in the first RHC.

## Discussion

We report here a case of leflunomide-induced PAH in which PAP and right heart function improved significantly after discontinuation of the drug. Although improvement of the patient’s symptoms and PAP may have been related to the suspension of leflunomide, other possible contributing factors should also be considered. First, NS itself can lead to PAH, although the etiology of PAH is still not clear. However, NS itself does not lead to a significant increase in PAP unless accompanied by some complications such as embolism (Du et al., 2004). Our patient’s severe PAH could not be attributed to NS itself. The mechanism of PAH induced by leflunomide may be related to its regulation of prostaglandin E2 (PGE2) metabolism. A low concentration of PGE2 induces the contraction of pulmonary arteries through E prostanoid 3 receptor (EP3). EP3 expression is decreased in PA smooth muscle cells and PAs in response to hypoxia (Lu et al., 2015). Chronic hypoxia causes a decrease in EP3, leading to PA remodeling and ultimately PAH. It was also reported that A771726, the active metabolite of leflunomide, can inhibit cyclooxygenase 2 (COX) directly to inhibit production of PGE2 (Burger et al., 2003). Therefore, it is likely that leflunomide caused PAH in our patient by decreasing the production of PGE2.

Initial combination therapy with targeted drugs for PAH such as macitentan and tadalafil have been shown to significantly improve MPAP, PVR, exercise tolerance, and functional parameters in newly diagnosed, treatment-naï ve patients (Marinescu et al., 2020). Four cases of PAH treated with leflunomide for rheumatoid arthritis (RA), psoriatic arthritis, and undetermined CTD were reported; all of the patients stopped leflunomide and 3 out of 4 received targeted PAH therapy, with all patients showing improvement of clinical symptoms and hemodynamic parameters (Coirier et al., 2018). Two other cases receiving leflunomide treatment for RA had favorable outcomes after suspending their drug treatment (Martinez Taboada et al., 2004; Alvarez et al., 2012). Thus, the significant improvement in systolic pressure of the RV in our patient suggests that suspension of leflunomide can improve PAH.

In summary, although mechanisms of drug-induced PAH are known and targeted PAH therapies have been broadly adopted, it is still important to exclude risk factors. PAH has been added to the leflunomide package insert as a special warning and precaution (Coirier et al., 2018); therefore, leflunomide should be discontinued as soon as possible in patients with NS who already have PAH and are taking leflunomide, after ruling out other causes of PAH without additional information regarding its pathogenesis.

## Data Availability

The original contributions presented in the study are included in the article/supplementary material, further inquiries can be directed to the corresponding author.

## References

[B1] Alvarez P. A. Saad A. K. Flagel S. Mazzocchi O. Blanco M. V. (2012). Leflunomide- induced pulmonary hypertension in a young woman with rheumatoid arthritis: A case report. Cardiovasc. Toxicol. 12 (2), 180–183. 10.1007/s12012-012-9153-3 22270725

[B2] Burger D. Begué -Pastor N. Benavent S. Gruaz L. Kaufmann M. T. Chicheportiche R. (2003). The active metabolite of leflunomide, A77 1726, inhibits the production of prostaglandin E(2), matrix metalloproteinase 1 and interleukin 6 in human fibroblast-like synoviocytes. Rheumatol. Oxf. 42 (1), 89–96. 10.1093/rheumatology/keg038 12509619

[B3] Coirier V. Lescoat A. Chabanne C. Fournet M. Coiffier G. Jouneau S. (2018). Pulmonary arterial hypertension in four patients treated by leflunomide. Jt. Bone Spine 85 (6), 761–763. 10.1016/j.jbspin.2017.12.014 29329993

[B4] Du Z. D. Cao L. Liang L. Chen D. Li Z. Z. (2004). Increased pulmonary arterial pressure in children with nephrotic syndrome. Arch. Dis. Child. 89 (9), 866–870. 10.1136/adc.2003.039289 15321868 PMC1763209

[B5] Galiè N. Humbert M. Vachiery J. L. Gibbs S. Lang I. Torbicki A. (2016). 2015 ESC/ERS guidelines for the diagnosis and treatment of pulmonary hypertension: The joint task force for the diagnosis and treatment of pulmonary hypertension of the European society of cardiology (ESC) and the European respiratory society (ERS) : Endorsed by: Association for European paediatric and congenital cardiology (AEPC), international society for heart and lung transplantation (ISHLT). Eur. Heart J. 37 (1), 67–119. 10.1093/eurheartj/ehv317 26320113

[B6] Lu A. Zuo C. He Y. Chen G. Piao L. Zhang J. (2015). EP3 receptor deficiency attenuates pulmonary hypertension through suppression of Rho/TGF- β 1 signaling. J. Clin. Investig. 125 (3), 1228–1242. 10.1172/JCI77656 25664856 PMC4362262

[B7] Marinescu D. Christiansen D. Thenganatt J. Granton J. T. (2020). Initial combination therapy with macitentan and tadalafil in pulmonary arterial hypertension: A retrospective cohort study. Am. J. Respir. Crit. Care Med. 201, A3827. 10.1164/ajrccm-conference.2020.201

[B8] Martinez Taboada V. M. Rodriguez Valverde V. Gonzalez Vilchez F. Armijo J. A. (2004). Pulmonary hypertension in a patient with rheumatoid arthritis treated with leflunomide. Rheumatol. Oxf. 43 (11), 1451–1453. 10.1093/rheumatology/keh328 15501999

[B9] Palasset T. L. Chaumais M. C. Weatherald J. Savale L. Jaïs X. Price L. C. (2021). Association between leflunomide and pulmonary hypertension. Ann. Am. Thorac. Soc. 18 (8), 1306–1315. 10.1513/AnnalsATS.202008-913OC 33502958

